# External Validation of the WORSEN Score for Prediction the Deterioration of Acute Ischemic Stroke in a Chinese Population

**DOI:** 10.3389/fneur.2020.00482

**Published:** 2020-05-29

**Authors:** Yicheng Xu, Yu Chen, Ruiwei Chen, Fei Zhao, Peifu Wang, Shengyuan Yu

**Affiliations:** ^1^Medical School of Chinese People's Liberation Army, Department of Neurology, Chinese People's Liberation Army General Hospital, Beijing, China; ^2^Department of Neurology, Aerospace Center Hospital, Beijing, China; ^3^Department of Radiology, Aerospace Center Hospital, Beijing, China

**Keywords:** acute ischemic stroke, early neurological deterioration, prediction, risk score, external validation

## Abstract

**Background:** Early neurological deterioration (END) has been recognized as a serious neurological complication after acute ischemic stroke. However, to date, the WORSEN score was the only one scoring system specifically developed to detect END events in acute ischemic stroke patients. The purpose of this study was to investigate the WORSEN score's utility in China, and to determine the potential predictors of END in acute stroke patients.

**Methods:** Consecutive patients with acute ischemic stroke admitted to the Department of Neurology, Aerospace Center Hospital between March 2015 to February 2017 were recruited into the study's cohort and divided into two groups: patients with and without END. END was defined as either an increase in two or more NIHSS points, an increment of at least one point in motor power or a description of fluctuating of clinical symptoms in medical reports during the first 7 days after admission. Severe END was defined as an increase of NIHSS ≥ 4 points from baseline during the first 7 days after admission.

**Results:** Three hundred fifty four patients with acute ischemic stroke were enrolled in the present study and 67.5% were male. END occurred in 90 of these patients and severe END occurred in 55 of these patients. Logistic regression analysis showed that an initial NIHSS score ≥8, diameter of infarction, striatocapsular infarction, and TOAST type of large arterial atherosclerosis were independent predictors for END. The area under the ROC curve (AUC) of the WORSEN score for the prediction of END was 0.80 (95%CI 0.75–0.84), with a sensitivity of 62.22%, a specificity of 88.26%, positive predictive values of 64.37% and negative predictive values of 87.27%. Meanwhile, the AUC of the WORSEN score for the prediction of severe END was 0.82 (95%CI 0.78–0.86), with a sensitivity of 70.91%, specificity of 83.95%, positive predictive values of 44.83% and negative predictive values of 94.01%.

**Conclusion:** END is a relatively common neurological complication in patients with acute ischemic stroke. Our findings showed that the WORSEN score had a good predictive value for identifying patients with END in a Chinese population. Moving forward, multi-center studies are required for further validations

## Introduction

Stroke is the second leading cause of death worldwide and is the cause of the highest disability-adjusted life-years lost in China (1). Early neurological deterioration (END), a relatively common neurological complication of stroke, occurs in 5–40% of acute ischemic stroke patients (2). Furthermore, it has been found that END is associated with poor outcomes (3, 4). Therefore, early detection of patients who are at risk of developing END is an important issue in clinical practice.

Many clinical and neuroimaging predictors have been associated with END. Simonsen et al. found that high blood glucose levels, the presence of large vessel disease, and a large perfusion lesion were associated with END (5). Yi et al. showed that patients with acute minor ischemic stroke with concomitant aspirin and clopidogrel resistance are at an increased risk for END (6) and Seners et al. reported that susceptibility vessel sign extension was an independent predictor of unexplained END in thrombolysed stroke patients (7).

However, when it comes to using scoring systems for assessing END in stroke patients, the WORSEN score is the only one that has been developed (Table 1). The WORSEN score (8), while not widely used, is based on definite clinical and imaging characteristics of stroke patients and is found to be a potentially valuable tool for detecting END in acute ischemic stroke patients. The purpose of this study was to investigate WORSEN scores' utility in our stroke population, and to determine the underlying predictor of END in acute stroke patients.

**Table 1:** WORSEN score.

**FACTORS**	
W	Wrong (poor) blood sugar control; HbA1c higher than 7.4% (1)
O	Old myocardial infarction (2)
R	Radiological findings: ICA occlusion (3) MCA M1 occlusion (2), striato-capsular infarction (1), pontine infarction (1)
S	Size of infarct (15–30 mm) (1)
E	Elevated LDL cholesterol level (>140 mg/dL) (1)
N	Neurological findings: initial NIHSS score higher than 8 (2)

HbA1c, glycated hemoglobin; ICA, internal carotid artery; LDL, low-density lipoprotein; MCA, middle cerebral artery; NIHSS, National Institutes of Health Stroke Scale.

*The numbers in parentheses indicate the number of points awarded for each findings*.

## Materials and Methods

### Subjects

This was a retrospective, observational study. Consecutive acute ischemic stroke patients admitted to the Department of Neurology, Aerospace Center Hospital between March 2015 and February 2017 were assessed. Patients were eligible for inclusion if they were 18 years or older, had a diagnosis of acute ischemic stroke defined in accordance with the World Health Organization criteria (9), and had symptom onset within 48 h. The diagnosis was verified by brain magnetic resonance imaging (MRI). Exclusion criteria of this present study were as follows: (1) age <18 years; (2) transient ischemic attack, intracerebral hemorrhage, subarachnoid hemorrhage, or brain tumors; (3) patients who accepted intravenous thrombolysis and(or) mechanical thrombectomy; (4) early discharge; (5) lack of data. This study was approved by the Ethics Committee of our hospital in accordance with the principles stated in the Declaration of Helsinki. Patient informed consent for inclusion in this study was waived.

### Clinical Variables

For all patients, we collected the following clinical information: age; sex; disease history, such as hypertension, diabetes mellitus, dyslipidemia, smoking history, atrial fibrillation, coronary artery disease, stroke, and transient ischemic attack; NIH stroke scale (NIHSS) scores (10) at baseline; initial blood pressure; stroke subtype, according to Trial of Org10172 in Acute Stroke Treatment (TOAST) criteria (11). Laboratory blood tests, including full blood counts, fasting blood glucose, hemoglobin A1c (HbA1c), triglycerides, high-density lipoprotein cholesterol, low-density lipoprotein cholesterol, uric acid and homocysteine were also collected. The score of WORSEN was calculated according to Miyamoto et al. (8).

### END and Severe END Definition

In order to draw attention to and encourage action to be taken in clinical practices, END was defined as an increase in two or more NIHSS points, an increment of at least one point in motor power, or description of fluctuating of clinical symptoms in medical reports during the first 7 days after admission (12–14). Severe END (15) was defined as an increase of NIHSS ≥ 4 points from baseline during the first 7 days after admission. Patients were classified into 2 groups according to the presence or absence of END.

### Statistical Analysis

Continuous variables were presented as the means (standard deviation, SD) or medians (interquartile range, IQR). Comparisons between the two groups were performed using Student's *t* test or the Mann-Whitney *U* test after testing for normality. The χ^2^ test or Fisher's exact test was conducted to analyze categorical variables. Logistic regression analysis was performed to evaluate variables' influence on END, adjusting for baseline variables when a *p* < 0.1 was found in the univariate analysis. We calculated the sensitivity and specificity of different levels of WORSEN scores for prediction of END (severe END) by using receiver operating characteristic (ROC) curves. All testing was two-tailed, and *p* < 0.05 were considered to be statistically significant. All data were analyzed using SPSS (Version 22.0, Windows, SPSS Inc., Chicago, IL).

## Results

**Figure 1:**
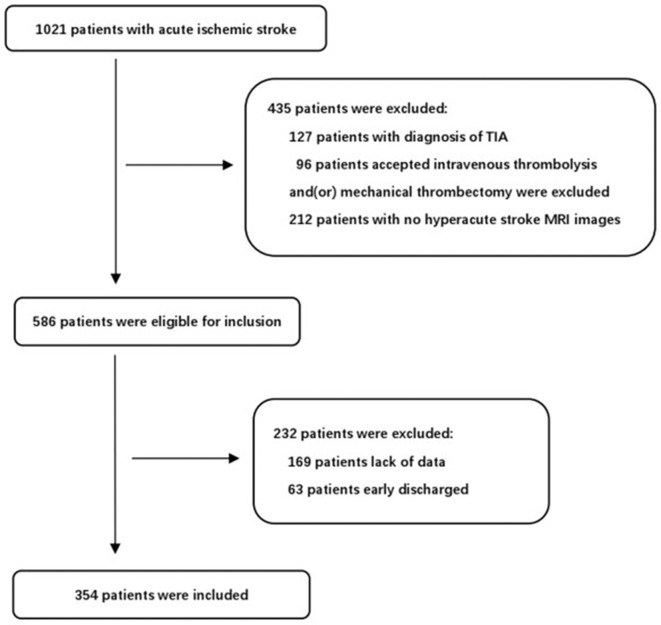
Flow chat of the patients included in the present study.

During the study period, 1,021 consecutive patients with acute ischemic stroke were potentially eligible for our study. After excluding patients that fit the exclusion criteria, 354 consecutive patients were finally included in the present study (Figure 1). The mean age was 64.2 ± 12.8 and 239 patients (67.5%) were male. END occurred in 90 of these patients (25.4%) and severe END occurred in 55 (15.5%). We identified the reasons for severe END in the 55 patients: four patients were due to hemorrhage transformation, seven patients due to malignant edema, ten patients due to recurrent stroke, seven patients due to medical complications, and 27 patients without a clear mechanism. We reported the demographics, baseline clinical characteristics and outcomes in patients with and without END (Table 2). There were statistically significant differences between the two groups (*P* < 0.05) in coronary disease, stroke etiology, percentage with an initial NIHSS score≥8, white blood count (WBC), neutrophil percentage, uric acid levels, striatocapsular infarction, diameter of infarct, and WORSEN score. However, there were no significant differences in other characteristics between the two groups. As expected, patients with END had worse modified Rankin Scale(mRS) scores at discharge than patients without.

**Table 2:** The baseline demographics, clinical characteristics, and outcome between the patients with and without END.

**Variables**	**With END**	**Without END**	***P***
	**(*n* = 90)**	**(*n* = 264)**	
Age, years	66.0 ± 13.5	63.6 ± 12.5	0.119
Male (%)	58 (64.4%)	181 (68.6%)	0.471
**VASCULAR RISK FACTORS (%)**			
Hypertension	63 (70.0%)	162 (61.2%)	0.142
Diabetes mellitus	33 (36.7%)	80 (30.2%)	0.807
Hypercholesterolemia	21 (23.3%)	50 (18.9%)	0.263
Smoking	40 (44.4%)	133 (50.4%)	0.284
Coronary disease	24 (26.7%)	35 (13.3%)	0.003
**Clinical Data**			
Systolic blood pressure (mmHg)	146.22 ± 22.70	147.09 ± 22.36	0.752
Diastolic blood pressure(mmHg)	84.53 ± 13.40	84.24 ± 13.37	0.859
Initial NIHSS score ≥ 8	49 (54.4)	25 (9.5)	<0.001
Stroke etiology (%)			<0.001
Large artery atherosclerosis	60 (66.7%)	108 (40.9%)	
Small artery occlusion	6 (6.7%)	69 (26.1%)	
Cardioembolism	16 (17.8%)	21 (8.0%)	
Others	4 (4.4%)	3 (1.1%)	
Unknown	4 (4.4%)	63 (23.9%)	
**LABORATORY AND IMAGING VARIABLES**			
White blood cell count (*10^9^/L)	7.42 ± 1.91	6.82 ± 1.78	0.007
Neutrophil percentage (%)	69.7 ± 10.8	64.3 ± 10.5	<0.001
Total cholesterol (mmol/L)	4.69 ± 1.39	4.99 ± 4.31	0.523
Triglyceride (mmol/L)	1.74 ± 1.28	2.11 ± 1.63	0.053
High-density lipoprotein (mmol/L)	0.75 ± 0.20	0.82 ± 0.62	0.355
Low-density lipoprotein (mmol/L)	2.64 ± 1.03	2.57 ± 0.81	0.500
Glucose (mmol/L)	8.31 ± 4.37	8.22 ± 4.10	0.872
Glycated hemoglobin	6.72 ± 1.47	6.72 ± 1.74	0.983
Uric acid (umol/L)	314.66 ± 91.61	337.83 ± 94.40	0.044
Homocysteine (mmol/L)	18.2 ± 12.90	17.47 ± 11.03	0.606
Striatocapsular infarction	45 (50.0%)	67 (25.4%)	<0.001
Pontine infarction	9 (10.0%)	40 (15.2%)	0.222
Diameter of infarct	31.02 ± 26.21	13.59 ± 10.85	<0.001
WORSEN score	3.20 ± 1.94	1.27 ± 1.24	<0.001
mRS score at discharge	3.34 ± 1.38	1.29 ± 0.80	<0.001

*NIHSS, National Institutes of Health Stroke Scale; mRS, modified Rankin Scale*.

Logistic regression analysis revealed that an initial NIHSS score ≥8 (odds ratio 2.64; 95%*CI* 1.13–6.19; *P* = 0.026*)*, diameter of infarction (odds ratio 1.03; 95%*CI* 1.01–1.05; *P* = 0.011), striatocapsular infarction (odds ratio 2.18; 95%*CI* 1.13–4.19; *P* = 0.020), and TOAST type of large arterial atherosclerosis (odds ratio 2.87; 95%*CI* 1.13–7.30; *P* = 0.027*)* were independently associated with END (Table 3).

**Table 3:** Logistic regression analysis for determinants of early neurological deterioration.

	***P* value**	**OR**	**95% *CI***
Initial NIHSS score≥8	0.001	4.91	2.50–9.64
Coronary disease	0.169	1.71	0.80–3.69
**STROKE ETIOLOGY**			
Others and Unknown	Reference		
Large artery atherosclerosis	0.027	2.87	1.13–7.30
Small artery occlusion	0.721	0.8	0.23–2.78
Cardioembolism	0.145	2.53	0.73–8.84
White blood cell count	0.223	1.13	0.93–1.36
Neutrophil percentage	0.143	1.03	0.99–1.06
Uric acid	0.09	0.99	0.99–1.00
Striatocapsular infarction	0.02	2.18	1.13–4.19
Diameter of infarction	0.008	1.03	1.01–1.05

*NIHSS, National Institutes of Health Stroke Scale; mRS, modified Rankin Scale*.

**Figure 2:**
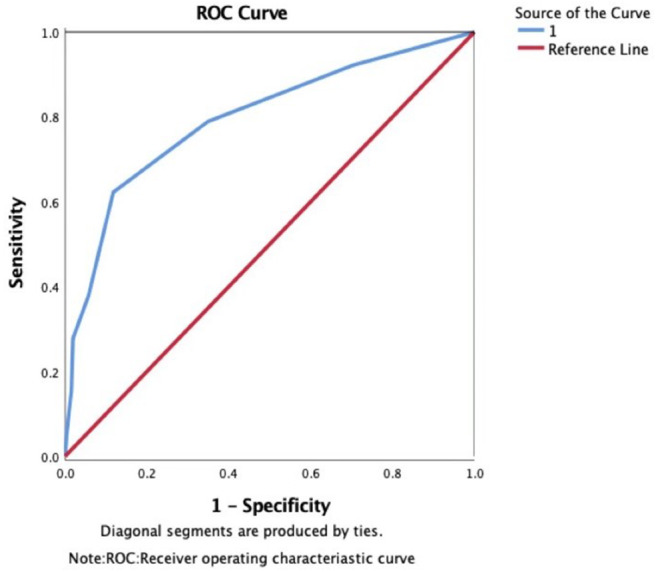
The predictive value of WORSEN score for END using ROC.

**Figure 3:**
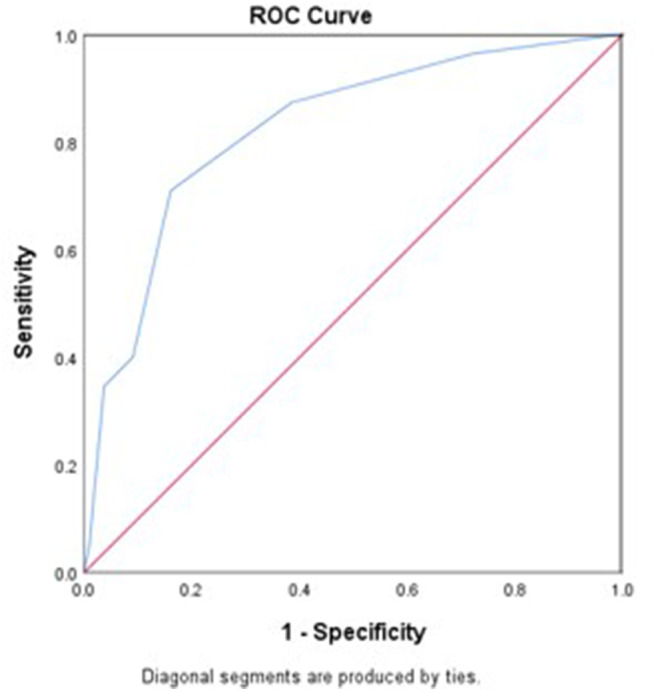
The predictive value of WORSEN score for severe END using ROC.

The area under the ROC curve (AUC) of the WORSEN score for the prediction of END was 0.80 (95%*CI* 0.75–0.84). Using Youden's index, the optimal cut point for a WORSEN score was 2, with a sensitivity of 62.22%, specificity of 88.26%, positive predictive values (PPV) of 64.37% and negative predictive values (NPV) of 87.27% (Figure 2). Meanwhile, the AUC of the WORSEN score for the prediction of severe END was 0.82 (95%*CI* 0.78–0.86); the optimal cut point for WORSEN score remained at 2 points, with a sensitivity of 70.91%, specificity of 83.95%, positive predictive values of 44.83% and negative predictive values of 94.01% (Figure 3). Table 4 shows the sensitivity, specificity, positive predictive value, negative predictive value and Youden's index for each WORSEN score in predicting severe END.

**Table 4:** Sensitivity (Se), specificity (Sp), positive predictive value (PPV), negative predictive value (NPV), and Youden index for each WORSEN score in predicting severe early neurological deterioration.

**WORSEN**	**Se**	**Sp**	**PPV**	**NPV**	**Youden**
**score**	**%**	**%**	**%**	**%**	**Index**
0	100	0	15.0		0
1	87.27	61.54	29.4	96.3	0.48
2	70.91	83.95	44.8	94	0.54
3	40	90.97	44.9	89.2	0.3
4	34.55	96.32	63.3	88.9	0.3
5	20	97.66	61.1	86.9	0.17
6	5.45	99	50	85.1	0.04
7	0	100		84.5	0

## Discussion

In our study, the incidence rates of END and severe END were 25.4 and 15.5% respectively. We found that an NIHSS score≥8, diameter of infarction, striatocapsular infarction, and TOAST type of large arterial atherosclerosis were independent predictors for END. WORSEN score showed the good predictive value in identifying patients with END after acute ischemic stroke in a Chinese population.

Although END has been well-recognized as a serious problem following acute ischemic stroke, the definition of END has been inconsistent among different studies and is still under constroversy (16). The reported incidence rate of total END in acute ischemic stroke patients without thrombolysis also has much uncertainty; ranging from 13.3 to 36.8% (2). Results from our study were in line with the two prior studies (6, 17), but were slightly higher than others (18, 19). This might be due to the fact that we adopted an increment in NIHSS scores≥2 points as the END definition. Siegler et al. had previously shown that the definition of END by an increase in NIHSS score of 2 or more points, as well as 4 or more points, were all associated with a poorer functional outcome at discharge (16). Our study showed that the mean mRS (3.34 ± 1.38) in the END group was significantly higher than in the non-END group (1.29 ± 0.80), which is consistent with the above findings. In the present study, we chose an increase in NIHSS score of 2 or more points as the definition of END because we wanted to advocate for early recognition and actions. However, some studies (2, 20) have pointed out that a small change in NIHSS score might be less meaningful and interrater dependent. Therefore, we also included an increase in NIHSS≥4 points for the severe END definition in our study.

With regard to the risk factors associated with END, hyperglycemia on admission (5), initial severity of stroke (19, 21, 22), mean platelet volume (17), metabolic syndrome (4), dehydration (23), presence of albuminuria (24) and fibrinogen (25), infarct size (19, 26) and proximal arterial occlusion (27) have been reported to be statistically significant in predicting END. Our findings that initial NIHSS score and infarct size are independently associated with END were consistent with previous studies, which may be explained by the fact that severe neurological impairment is a strong predictor of symptomatic intracranial hemorrhage (2) and malignant oedema (28). However, we did not find evidence that the blood sugar levels were associated with END, which is also in line with some other studies (17, 25, 28).

It is extremely important that clinicians are able to predicate the risk of END in patients with acute ischemic stroke in the early stages in order to choose an appropriate treatment method. Although many predictive models have been developed to project short-term or long-term functional outcomes of the disease and the risk of recurrence in patients (29–31), the WORSEN score (8) was the only one specifically developed for detection of END in acute ischemic stroke patients. Our study aimed to externally validate the WORSEN score in our population, and our findings indicated that it had a good predictive value for severe END with a sensitivity of 70.91%, specificity of 83.95%, and an the AUC of WORSEN score of 0.82 (95%*CI* 0.78–0.86).Fittingly, our findings were in line with prior studies.

Our study had several limitations. First, it was a single-center retrospective observational study. Patients with insufficient data for analysis were excluded and, despite the fact that included patients were consecutive, selection bias might have occurred and could have had a negative impact on our findings. Prospectively, large sample size and multi-center studies should be performed to confirm our findings.

Another limitation is in the various definitions of END. Despite the fact that we adopted the widely recommended definition of END and used logistic regression to build the predictive model, the possibility for residual confounding remains. Furthermore, although we excluded patients who accepted intravenous thrombolysis and (or) mechanical thrombectomy, we still could not eliminate the influence different treatments have on the outcomes of acute stroke patients. Finally, as the time of END has been recruited could be different for each patient, which might have also introduced bias.

In conclusion, END is a relatively common neurological complication in patients with acute ischemic stroke. Our study demonstrated that the WORSEN score had a good predictive power for identifying patients with END in a Chinese population. Moving forward, multi-center studies are required for further validate our findings.

## Data Availability Statement

The datasets for this article are not publicly available as data collection is still in progress. Requests to access the datasets should be directed to YX, yichengxu27@aliyun.com.

## Ethics Statement

The studies involving human participants were reviewed and approved by the study was approved by the Institutional Ethical Committee of Aerospace Center Hospital. The ethics committee waived the requirement of written informed consent for participation.

## Author Contributions

YX conceived the study, participated in the design, performed statistical analyses and drafted the manuscript. YC participated in the design, collected the data, and drafted the manuscript. RC performed statistical analyses and helped to draft the manuscript. FZ and PW participated in the statistical analyses. SY participated in the design and helped to revise the manuscript critically for important intellectual content. All authors read and approved the final manuscript.

## Conflict of Interest

The authors declare that the research was conducted in the absence of any commercial or financial relationships that could be construed as a potential conflict of interest.
